# Supplementary material for: Water regulates the residence time of Benzamidine in Trypsin
Source: Nat Commun. 2022 Sep 16;13:5438. doi: 10.1038/s41467-022-33104-3 (PMC9481606; doi:10.1038/s41467-022-33104-3)
Supplement: Supplementary file 1 — Supplementary information [file 41467_2022_33104_MOESM1_ESM.pdf]

Supplementary Information: Water regulates the residence time of Benzamidine in Trypsin

Narjes Ansari[1], Valerio Rizzi[1], and Michele Parrinello[1,*]

[1]Italian Institute of Technology, Via E. Melen 83, 16152, Genova, Italy
[*]michele.parrinello@iit.it

Supplementary Methods

Free energy simulations

For calculating the free energy surface of the system at T=300 K, we used 32 multiple walkers using OPES with `BARRIER` = 50 kJ mol^{-1} and `PACE` = 500. At first, we ran an equilibration where all the walkers contributed in generating the bias. Each walker was run for about 60 ns and the resulting bias potential reached the quasi-static regime. Then we decoupled the walkers and restarted them independently with the previously generated external potential as a bias. We ran these 32 independent simulations for around 100 ns each (see Supplementary Figure 2). We merge these trajectories into one that is about 3.2 μs long and use it to estimate the binding free energy.

For calculating average properties and their statistical error, we follow the same strategy of Ref. 1. For numerical stability, we filter out the outlier configurations where the funnel restraint, walls over the z and water CVs bias are larger than a threshold of 10 kJ mol^{-1}. We split the resulting trajectory into $N_\text{B} = 9$ blocks of ≈ 350 ns each. In each block i, we independently evaluate the binding free energy ΔF_i through Eq. 4 (see the main text). Every block has a different statistical weight given by $w_\text{i} = \sum_j e^{\beta V(j)}$ where index j runs over the configurations in the block and V is the bias potential.

The mean ΔF is then simply the weighted average over the blocks

$$\Delta F = \frac{\sum_{i=1}^{N_\text{B}} w_i \, \Delta F_\text{i}}{\sum_{i=1}^{N_\text{B}} w_i} \tag{1}$$

and its error is the weighted standard deviation

$$\sigma(\Delta F) = \sqrt{\frac{1}{N_\text{eff} - 1} \, \frac{\sum_{i=1}^{N_\text{B}} w_i \, (\Delta F_\text{i} - \Delta F)^2}{\sum_{i=1}^{N_\text{B}} w_i}}. \tag{2}$$

The effective sample size

$$N_\text{eff} = \frac{\left(\sum_{i=1}^{N_\text{B}} w_i\right)^2}{\sum_{i=1}^{N_\text{B}} w_i^2} \tag{3}$$

measures the the quality of the weighted block average by looking at the weight distribution in the blocks. In the ideal case of low correlation, $N_\text{eff} \to N_\text{B}$. We observe a $N_\text{eff} \approx 8.4$. In Supplementary Table 1, we show ΔF_i, w_i for each block and the average ΔF with its error.

For comparison, we also run a simulation where only z was biased for an analogous amount of total simulation time. The trajectories of the corresponding 8 walkers are in Supplementary Figure 3. Due to

the inefficiency of biasing only the z CV and not accelerating the water degrees of freedom, it is clear that sampling is severely slowed down as we do not observe any back and forth event. In Supplementary Figure 4a we show the free energy of binding from this simulation using a block average and a single block at different simulation times. The lack of convergence is again very clear. In Supplementary Figure 4b, we show the analogous plots from the converged simulation where both z and s_w are biased.

In Supplementary Figure 5, we show the binding free energy as a function of block number and time for both simulations. In the one where both z and s_w CVs are biased, the binding free energy strongly converges to a value of -6.3 kJ/mol. In the simulation where only z is biased, the free energy of binding is not converged. The data of its block average with the corresponding weights can be found in Supplementary Table 2. The weight distribution is very inhomogeneous as the blocks do not contain a sufficient sampling of the phase space. Such weight distribution indicates the lack of convergence and the unsuitability to perform a block average in this case. In fact, the resulting effective sample size $N_{\mathrm{eff}} \approx 2.0$, a much lower value than the ideal $N_B = 9$.

We performed an analogous strategy in the calculation of the enthalpy and entropy of binding. We run four different OPES simulations in the temperature range of 290 K $\leq$ T $\leq$ 330 K with a step of 10 K. We used the same CVs as in the room temperature simulation and the same OPES parameters and setup. For each temperature, we ran 8 multiple walkers and merged the resulting trajectories into one that is 2.5 μs long. We performed the same block analysis on each of the simulation and show the breakdown of the results in Supplementary Table 3. The final average ΔFs with their error are shown in Supplementary Table 4. Supplementary Figure 10 shows a plot of the ΔF as a function of temperature with the linear fit used for estimating the binding entropy.

Rate simulations

For the rate calculations, we use the newly developed approach OPES$_f$ [2]. We run in total 55 simulations using OPES$_f$ where we bias z and s_t with a `BARRIER` parameter of 22 kJ/mol, `PACE` of 100 steps and `EXCLUDED_REGION` $z > 6$ Å. The `EXCLUDED_REGION` is chosen given the knowledge of the free energy landscape from preceding simulations. One can see from the 2D FES in Figure 4a of the main text that the transition region is indeed located at $z > 6$ Å. In Supplementary Figure 6a, we show a trajectory of z in a typical OPES$_f$ simulation. In Supplementary Figure 6b, there is a 2D scatter plot of the same trajectory over the CVs z and s_t. Both plots are colored with the instantaneous value of the OPES bias, with black corresponding to the case where no bias is present. As required, the `EXCLUDED_REGION` (z > 6 Å) is unbiased.

For fitting the residence time data, we use the script from Ref. 3. In Supplementary Figure 13a, we show the cumulative probability of the transition rate from the simulations (red line) and fitted by an exponential (black line) using all the data. As discussed in the main text, the transitions occur according to a faster and a slower mechanism. We observed that 23 trajectories display the faster mechanism (see Supplementary Figure 13b), while 32 trajectories show the slower one (see Supplementary Figure 13c).

To ensure that the pathways that we observe between state B1 and I occur in unbiased simulations, we perform OPES$_f$ simulations with `EXCLUDED_REGION` $z > 4$ Å, `BARRIER` of 10 kJ/mol and PACE of 100 steps. For comparison, we bias both z and s_t and only z (see Supplementary Figure 14). In both cases, we observe both slow and fast mechanism events. In the former case, out of 7 simulations, 5 belong to the slow mechanism and 2 to the fast one. In the latter case, out of 13 simulations, 9 belong to the slow mechanism and 4 to the fast one. The proportion is analogous to the one of the full unbinding OPES$_f$ simulations.

Neural Network CVs training

The Deep-LDA strategy that we use in this paper is analogous to the one presented in Ref. 4, so we refer the interested reader to that paper for more details. A tutorial of Deep-LDA is present at the following link `https://github.com/luigibonati/data-driven-CVs`. Our NN architecture consists of a sequence of layers with $18, 12, 6, 4$ nodes, with the rectified linear unit as activation function. The descriptors set has 18 components ({G}, {H}, {V_i}). Training is performed by feeding the NN unbiased simulations about

60 ns long. One state is U, the other one starts from the initial configuration used in Ref. 5 which we later realized presents a reservoir region with one water molecule less than what we call state B in the paper. The number of configurations per state used in the training is 100000. These configurations are divided in batches of 512 elements. We use Deep-LDA parameters $\lambda = 0.05$, $\alpha = 2/\lambda$, $\gamma = 10^{-5}$. The model is optimised with the ADAM optimizer [6] using a learning rate of $2.5 \cdot 10^{-5}$.

The Deep-TICA strategy follows Ref. 7 and a tutorial is available at the following link https://github.com/luigibonati/deep-learning-slow-modes. The NN architecture is made of a sequence of 4 layers with the rectified linear unit as activation function. The descriptors set is the same as the one used in Deep-LDA, while the training trajectory is the OPES simulation used for estimating the FES at 300 K. We use the ADAM optimizer [6] with a 1.0×10^{-4} learning rate and the loss function included two TICA eigenvalues. We train the Deep-TICA model using a lag-time of 0.07 and use the resulting CV in the analysis in the rate calculations.

Volume of the B and B′ states binding pose

The presence of one water molecule in the V9 position determines that the system lies in state B or in state B′. To evaluate the volume of the binding pose in these two states, we extract configurations in each of the two states from an ensemble of OPES_f calculations lasting about 350 ns. To discriminate the two states, we set a threshold of $s_\text{t} = -0.2$. Using the coordinates of the α-Carbon atoms, shown in Supplementary Figure 7a as white spheres, we build a convex hull to estimate the volume of this region in the two cases. Supplementary Figure 7b shows that the volume of state B is on average around 5 % larger than state B′.

Ionic density analysis

In Ref. 8, Roussey and Dickson find that the ionic density plays a relevant role in ligand (un)binding. Our simulations includes 7 Cl^- ions. To evaluate the ionic importance in our system, we measure their presence in the vicinity of the binding site and the ligand itself. We first count the number of Cl^- ions within 5 of the Carbon atoms of the carboxylate group in residue Asp189 in our 3.2 μs biased trajectory. We do not observe any presence of Cl^-, as the probability of finding a negative ion in the binding pose is severely hindered by the fact that residue Asp189 has a negative charge. On the other hand, the ligand Benzamidine presents a partial positive charge on its Carbon atoms. Analogously, we count the number of Cl^- ions within 5 of the Carbon atom of the Benzamidine amidine group and observe the presence of an ion within the cutoff only in 3.6×10^{-5}% of the trajectory. This observations lead us to believe that the ions do not play a relevant role in our ligand binding system. In other systems where the ions are more relevant, it would indeed be interesting and beneficial to capture them in a CV and accelerate them.

1D FES along Deep-TICA CV

As shown in Figure 3 in the main text, the Deep-TICA CV is able to identify two minima corresponding to states B and B′ which are not well resolved in the space of Deep-LDA. To calculate the energy difference between these two states, we run two OPES simulations using z and s_t CVs, trapping the ligand inside (holo) and outside (apo) of the binding pocket. Supplementary Figure 8 shows the 1D FES of those simulations. In both cases, state B is around 12 kJ/mol more stable than state B′.

Transition state region along z and Deep-TICA CVs

We analyze the transition state region $z > 6$ Å by taking the unbiased portion of OPES_f simulations and calculating the 2D FES along z and water CVs s_w (Supplementary Figure 9a) and s_t (Supplementary Figure 9b). A typical transition path is shown with a dashed white line. The Deep-LDA CV has no projection along the path, while the Deep-TICA CV has a more prominent role.

Binding enthalpy and entropy

In Supplementary Figure 10, we show the binding free energy as a function of temperature with its standard deviation. The corresponding data is reported in Supplementary Table 4. The binding enthalpy and entropy can be extracted by a linear fit procedure, using the relationship $\Delta F = \Delta U - T\Delta S$. To estimate

the error on ΔU and $T\Delta S$ that we show in Table 1, we use a bootstrap procedure like the one from Ref. 9 that we used in Ref. 1. We produce 100 sets of $\Delta F(T)$ by randomly generating free energy values within the error range. For each of these sets, we perform a linear fit procedure and determine the corresponding ΔU and $T\Delta S$. Their error is the standard deviation on these estimates.

Ranking of descriptors in Deep-LDA and Deep-TICA CVs

In order to compare the relative importance of the different water descriptors **d** in the NN CVs, we use the derivative ranking method introduced in Ref. 4. For the Deep-LDA CV s_{w}, we calculate separately the descriptor ranking in state B and U, while for the Deep-TICA CV s_{t} we use all data.

As shown in Supplementary Figure 11a and b, the distribution of weights for s_{w} is quite spread out over the whole descriptor set which indicates the ability of the Deep-LDA CV to capture the complex non-local behavior of water. We stress upon the importance of finding and including relevant long-lived water molecules in the descriptor set used in the Deep-LDA training. For instance, a reduced descriptor set that discriminate states well is not a sufficient condition to produce an effective CV to bias the system. Knowledge of the system and its slow degrees of freedom is essential. If a CV classifies well but does not include information about important slow degrees of freedom of the system, it would be a good classifier but would not be able to push the system effectively between such states.

Interestingly, high weights of descriptors in the reservoir region reveal the important role of water in this region for both the binding and unbinding process (see panel a and b in Supplementary Figure 11. As shown in Supplementary Figure 11a, in state B, descriptors G and V16 have higher weights, which shows to push ligand toward outward one needs to amplify water in this region. While for state U (see panel b in Supplementary Figure 11), descriptors along the path are equally important for binding process.

In the case of Deep-TICA, as shown in Supplementary Figure 11 around 50 % of the weight over V9, shows the significant role of water in this region as a slowest degree of freedom in the ligand unbinding process. In fact, the presence of water in this region acts as a switch to activate the unbinding process.

Orientational analysis of the water molecules

Our water CVs are trained on information that is based on water density. Here we discuss whether including orientation information on the water molecules might improve our results.

The lifetime of buried water molecules ranges from ns to μs, while molecular rotations occur on a much faster timescale, typically ps. This observation indicates that the slowest water modes lie in the translation of water molecules towards and away from their long-lived positions rather than in their orientation. To corroborate this claim, we perform a water analysis inspired by the work of Gelenter et al. [10] where one evaluates the non-uniformity of orientation of water molecules by estimating the entropy parameter $\Delta\Gamma$

$$\Delta\Gamma = \sum_{i=0}^{N_{\mathrm{bin}}} P(\cos(\theta)) \ln[P(\cos(\theta))] + \ln[N_{\mathrm{bin}}] \tag{4}$$

where θ is the angle between a water molecule's dipole moment and a fixed axis z, $P(\cos(\theta))$ is a probability density and N_{bin} is the total number of bins in θ. Higher $\Delta\Gamma$ value correspond to more ordered water and a higher orientational preference.

We perform this analysis over water in the vicinity of each hydration spot $\{\mathrm{V}_i\}$ in two sets of trajectories, unbiased and biased, where both state B and U are visited. We consider water molecules located within 2 Å of $\{\mathrm{V}_i\}$. θ is the angle between their dipole moment and the binding axis z and we discretize it with a spacing of 0.1 radians. In Supplementary Figure 12, we show the value of $\Delta\Gamma$ over unbiased (state B and U) and biased simulations and observe an analogous trend between the two. This indicates that the biased simulations reproduce the water ordering from unbiased simulations, even if no explicit information about the water orientation in present in the biased CVs.

Supplementary Figures

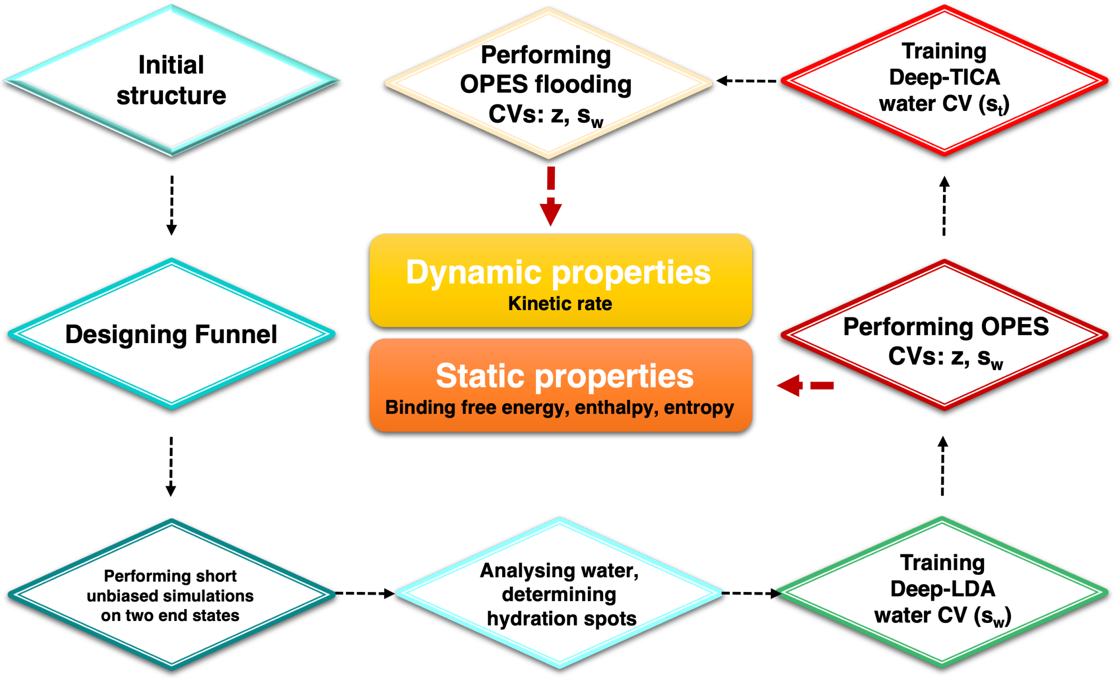

Supplementary Figure 1: **Flowchart of the method.**

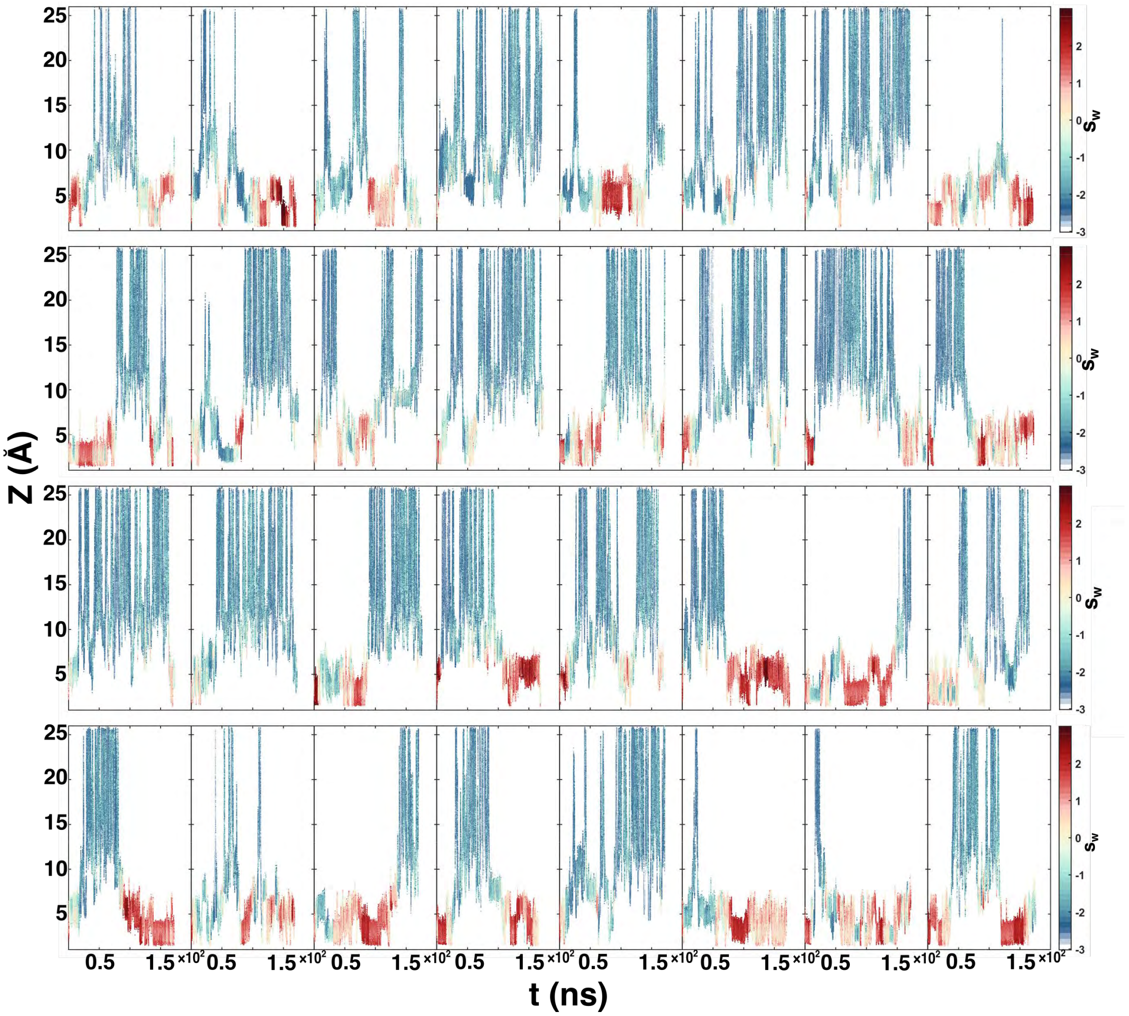

Supplementary Figure 2: **OPES trajectories biasing z and s_w.** Dynamics of z in the 32 walkers OPES simulation at 300 K where both z and s_w are biased. The plots are colored with the instantaneous value of s_w.

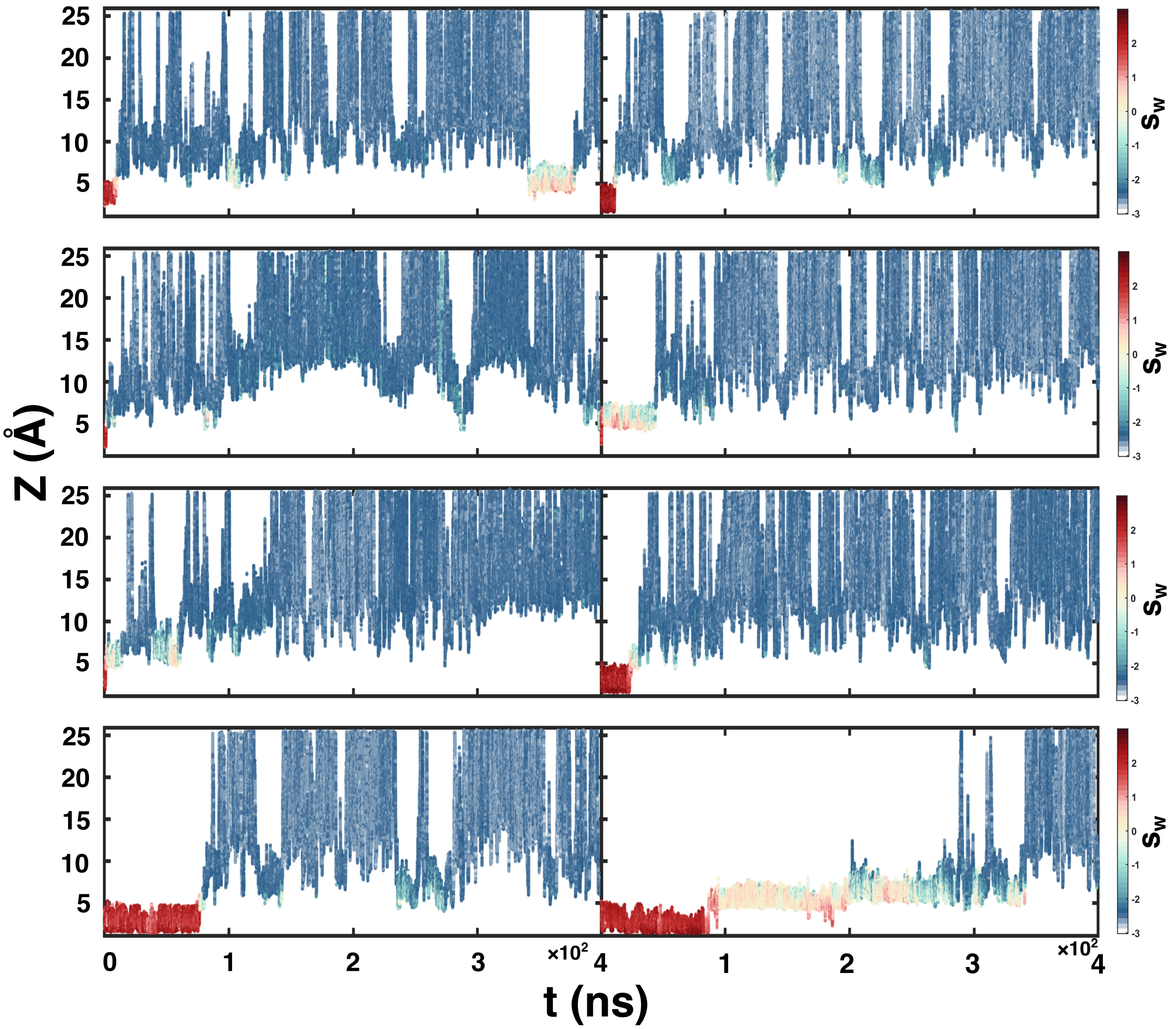

Supplementary Figure 3: **OPES trajectories biasing only** z**.** Dynamics of z in the 8 walkers OPES simulation at 300 K where only z is biased. The plots are colored with the instantaneous value of s_w.

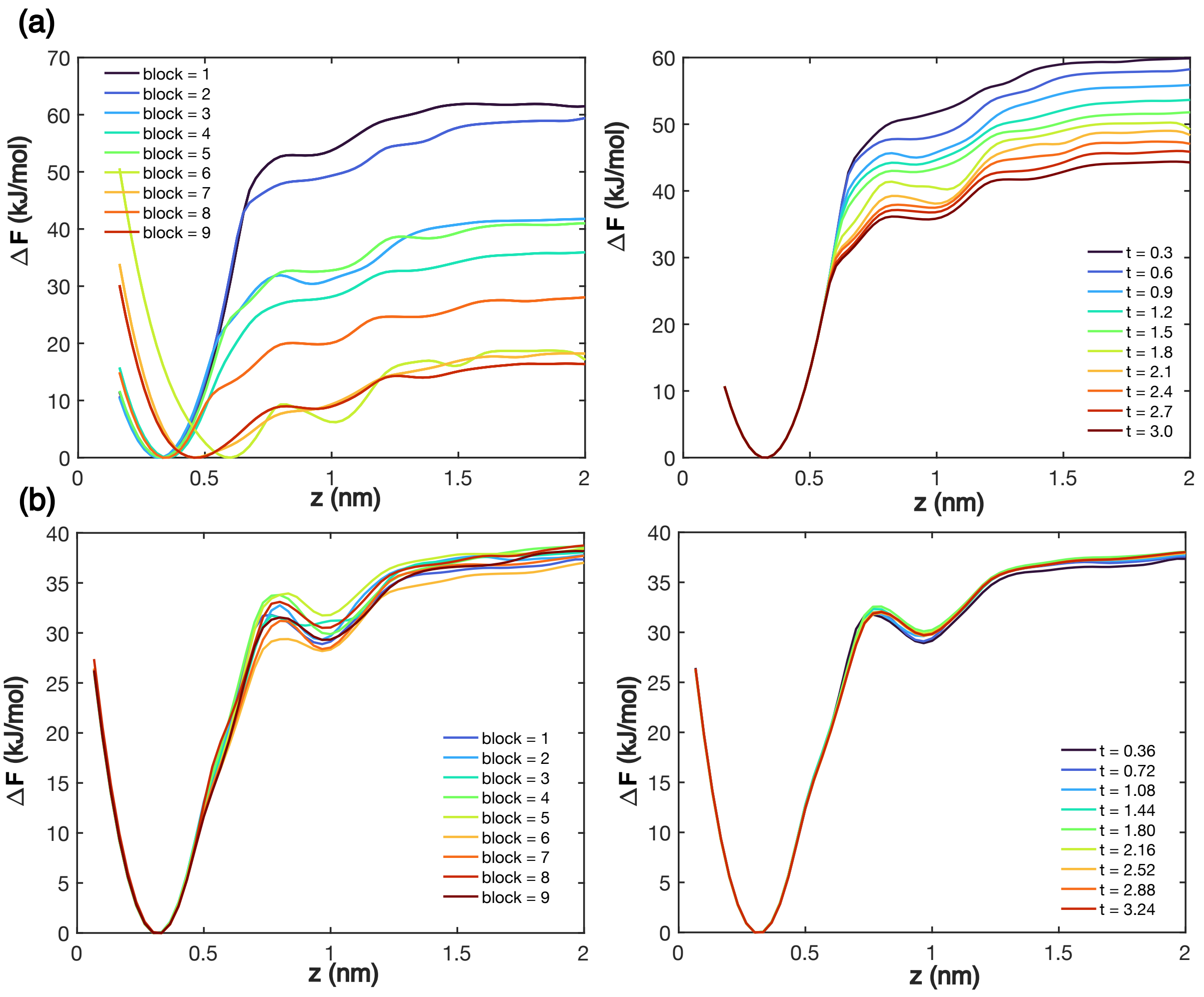

Supplementary Figure 4: **Free energy surface comparison using different CVs.** FES calculated from simulations where we biased (a) only z and (b) both z and s_{w}. In both panels, the left hand side shows FESs from block analysis and the right hand side FESs evaluated at increasing simulation time.

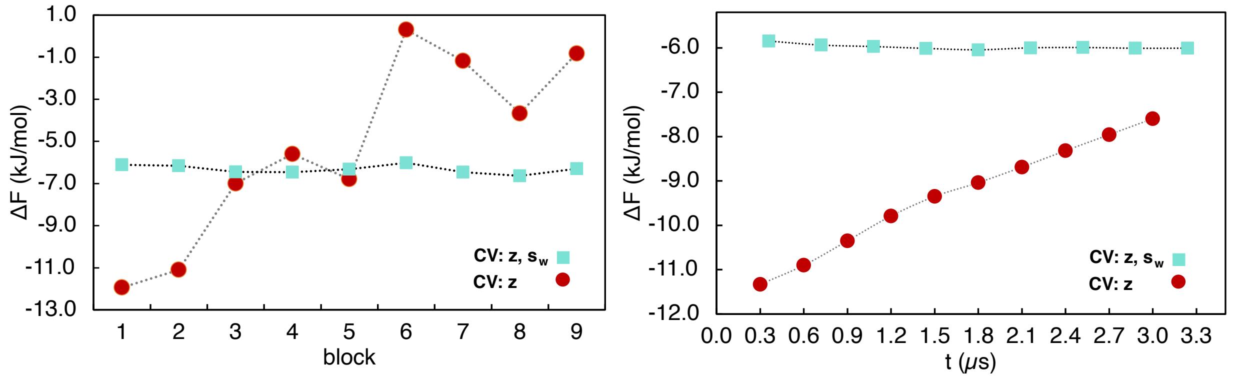

Supplementary Figure 5: **Binding free energy comparison using different CVs.** Binding free energy ΔF (kcal mol^{-1}) calculated from simulations where we biased (a) only z and (b) both z and s_{w}. In both panels, the left hand side shows the value from block analysis and the right hand side FESs evaluated at increasing simulation time.

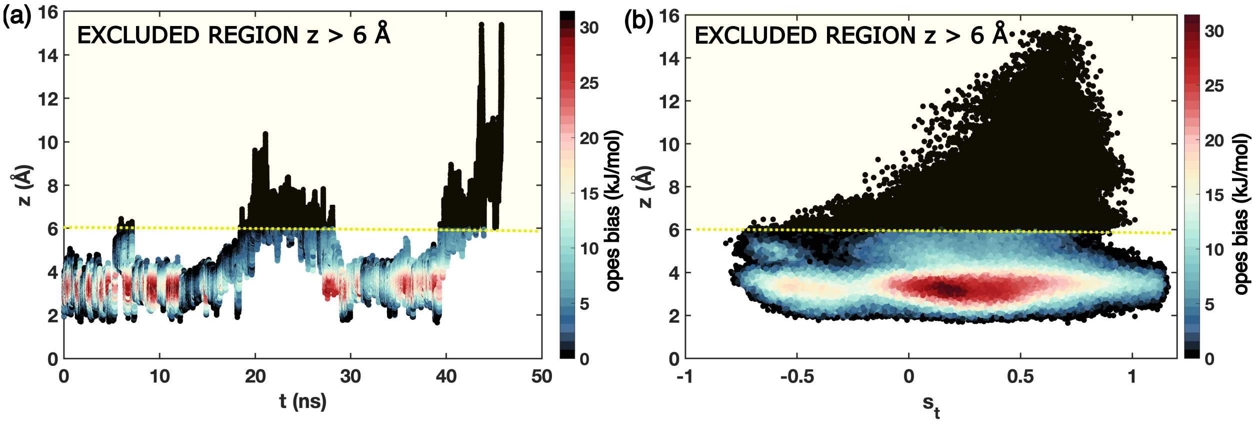

Supplementary Figure 6: **A representative trajectory of OPES$_{\mathrm{f}}$** along (a) z and time, (b) z and s_{t}. The transparent yellow box shows the EXCLUDED REGION ($z > 6$ Å), where the opes bias is equal to zero.

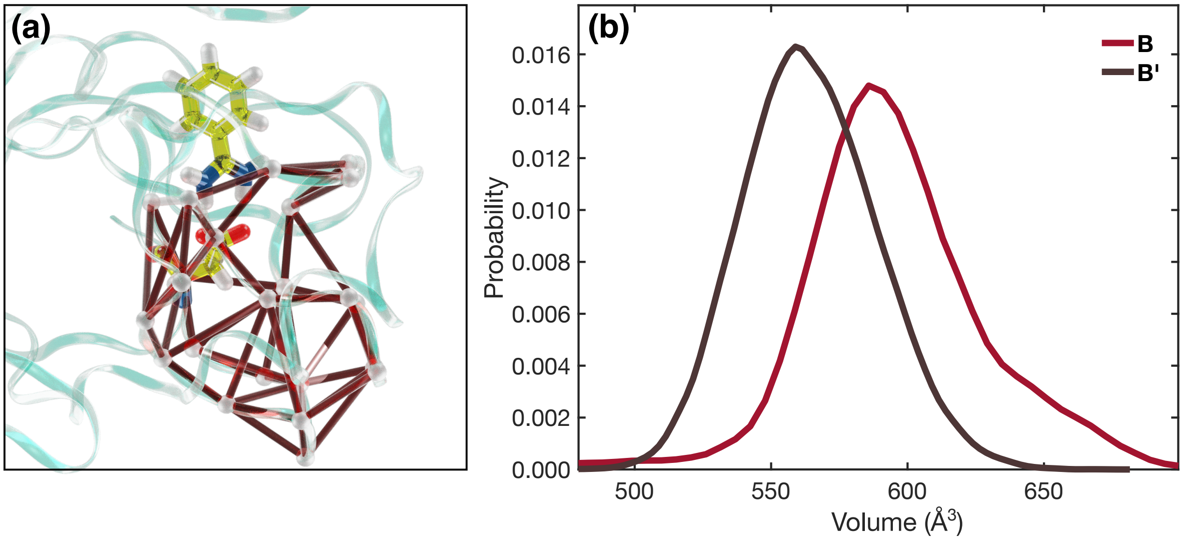

Supplementary Figure 7: **Binding pose volume estimation.** (a) Schematics of the convex hull surface around the binding pose of Trypsin. The α-Carbon atoms of the residues located in this region are represented by small white spheres. (b) Volume probability distribution for convex hulls on trajectories of states B (red) and B′ (gray).

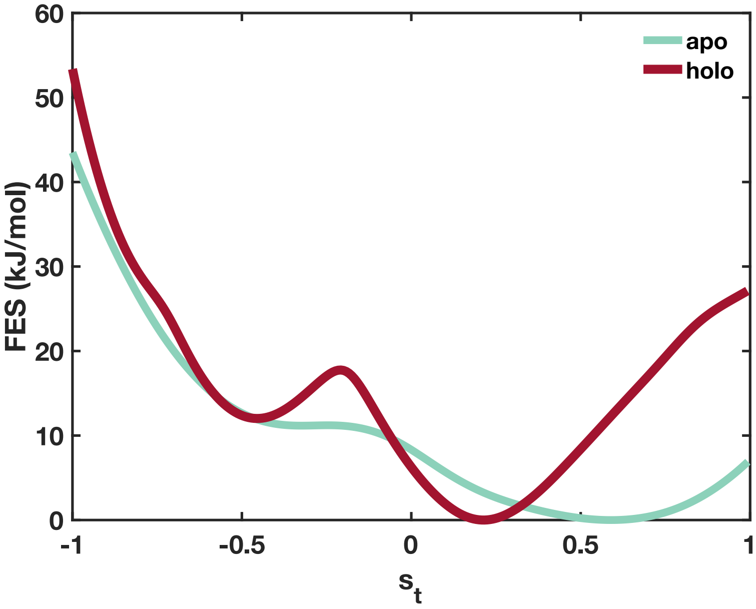

Supplementary Figure 8: **Deep-TICA FES projection.** 1D FES along s_t for the apo and holo forms of the Trypsin-Benzamidine system.

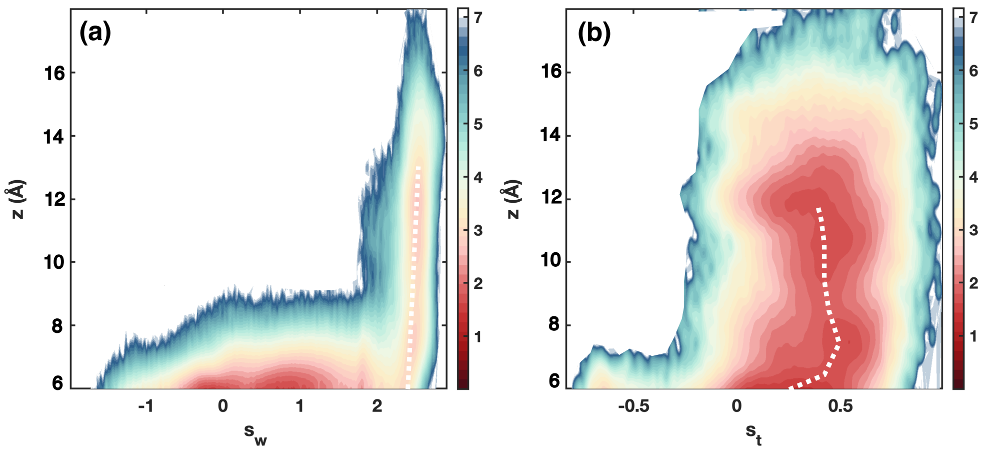

Supplementary Figure 9: **Transition state region.** 2D FES along z and (a) Deep-LDA and (b) Deep-TICA water CVs for data z > 6 Å

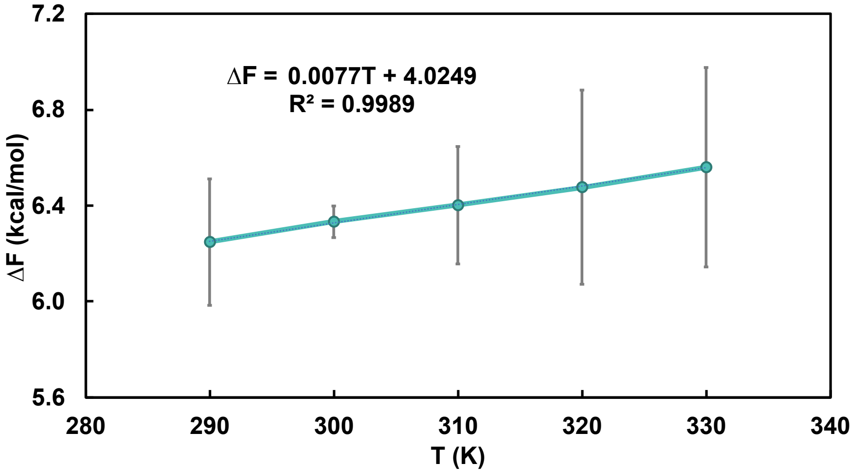

Supplementary Figure 10: **Binding free energy as a function of the temperature.** The slope and intercept of the plot correspond to entropy and enthalpy of binding, respectively.

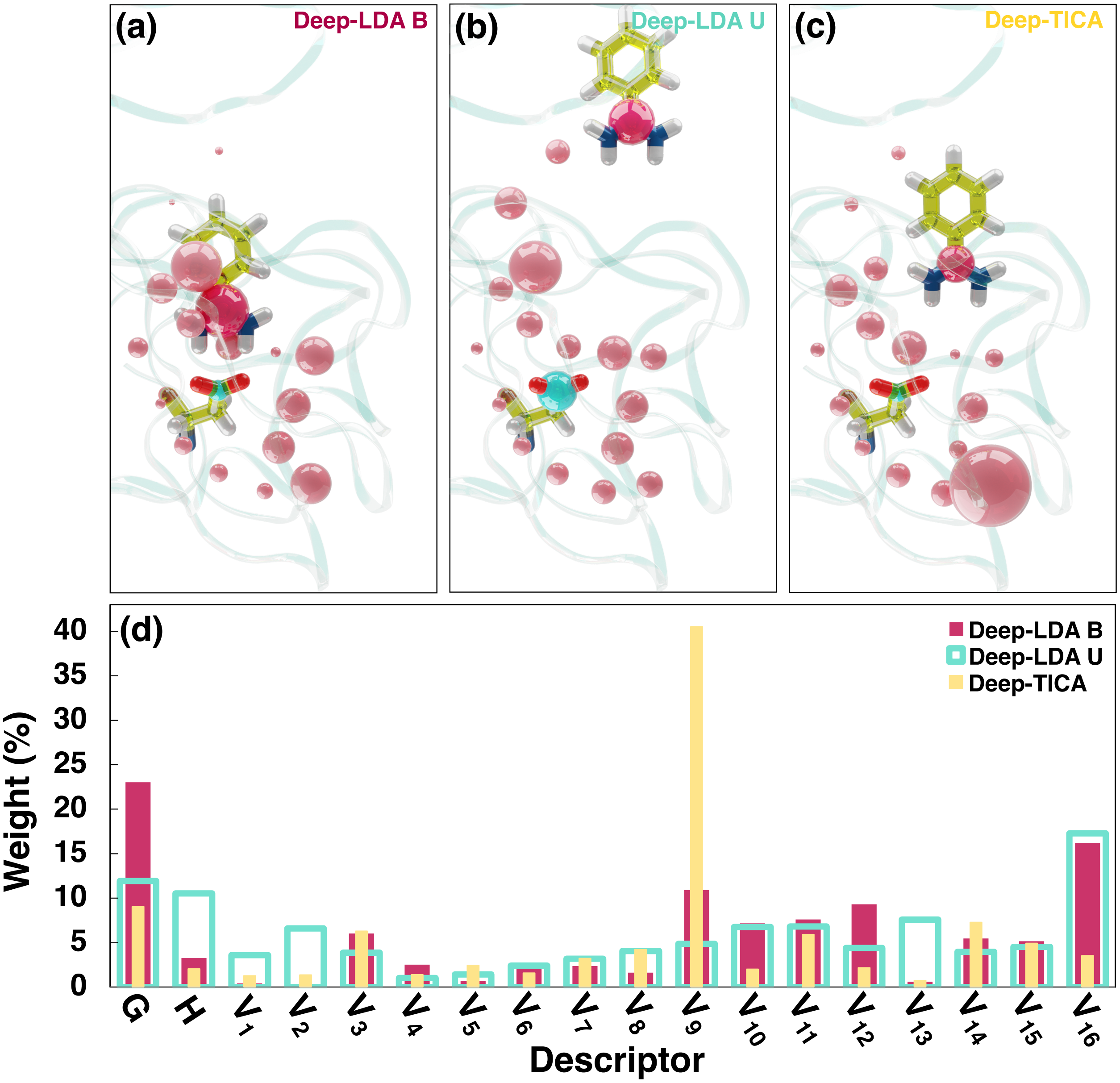

Supplementary Figure 11: **Descriptors relative weights.** Derivative-based ranking of descriptors for the water CV Deep-LDA in (a) B and (b) U states. (c) Same as panel (a) and (b) for water CV Deep-TICA. In this case we use all the data for ranking. (d) Histogram of the weights.

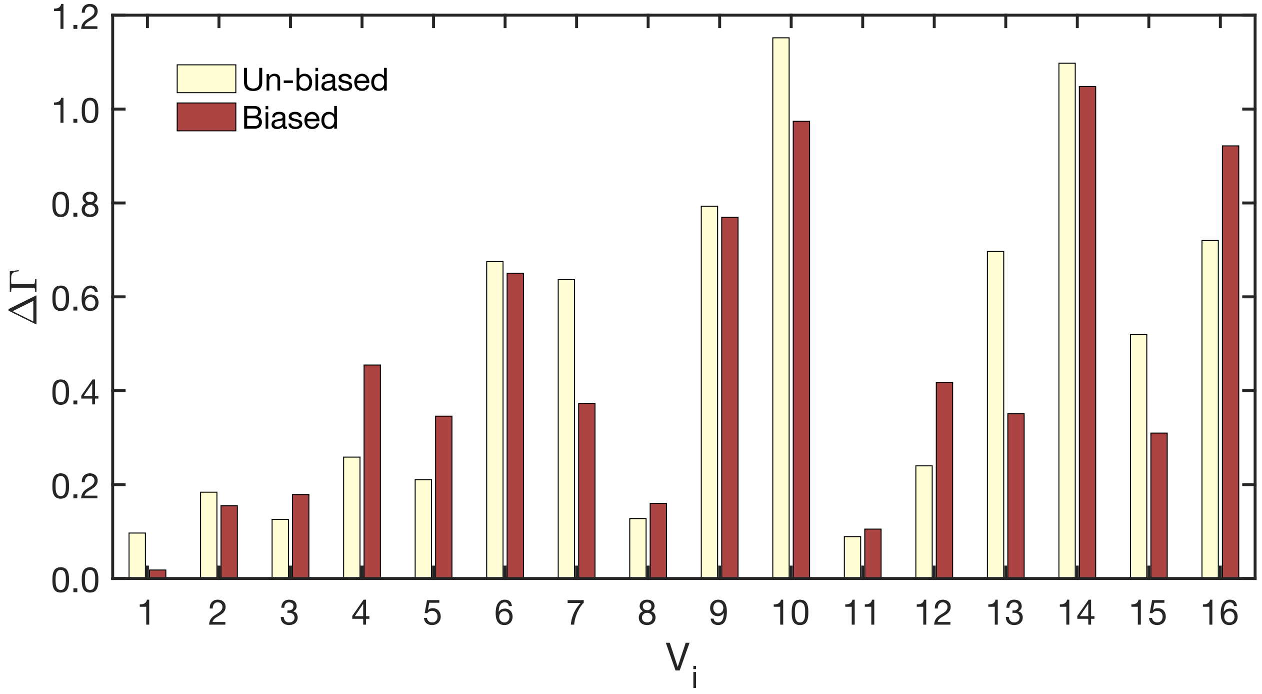

Supplementary Figure 12: **Entropy parameter** estimated over biased (red) and unbiased trajectories (yellow) on water molecules in the vicinity of the 16 $\{V_i\}$ hydration spots.

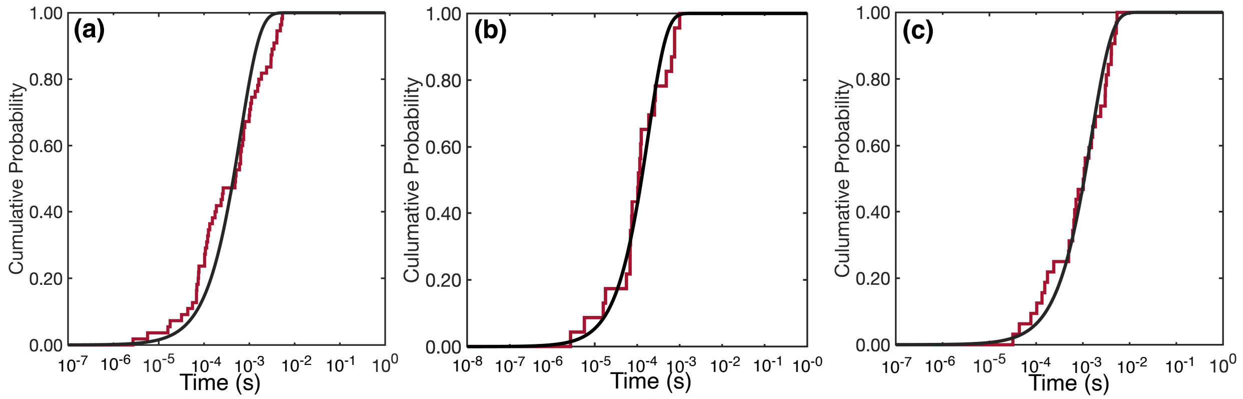

Supplementary Figure 13: **Unbinding rate Poissonian fits.** Cumulative distributions of the rate directly evaluated from the simulations (red line) and fitted as a Poisson process (black line) using (a) all trajectories, (b) the trajectories belonging to the slower and (c) the faster mechanism.

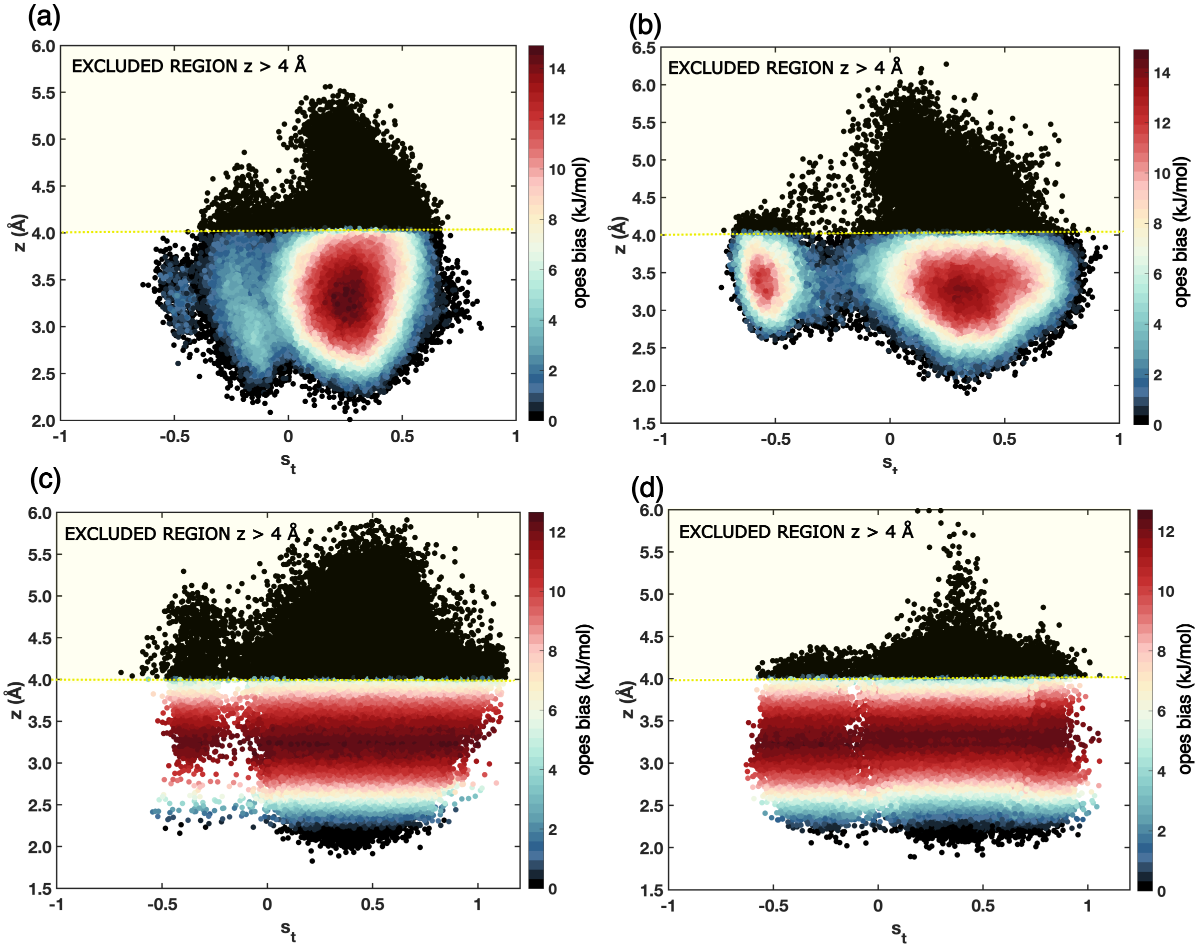

Supplementary Figure 14: **Pathways to state I.** Representative OPES$_f$ trajectories with EXCLUDED REGION $z > 4$ Å where (a,b) z and s_t are biased and (c,d) only z is biased. The transparent yellow box shows the EXCLUDED REGION where the opes bias is equal to zero and the plots are colored with the bias. Trajectories (a,c) display the faster mechanism and (b,d) the slower one.

Supplementary Tables

Supplementary Table 1: **Binding free energy breakdown at T=300 K.** Binding free energy ΔF (kcal mol^{-1}) and its corresponding statistical weight w (a.u.) in every simulation block of the calculations biasing z and s_{w}.

Block	CVs ΔF	z, s_{w} w
1	6.11	100
2	6.16	100
3	6.45	140
4	6.46	160
5	6.32	100
6	6.02	69
7	6.46	130
8	6.64	110
9	6.30	73
all	**6.36** $\pm$ 0.07	

Supplementary Table 2: **Binding free energy block analysis using only z.** Binding free energy ΔF (kcal mol^{-1}) and its corresponding statistical weight w (a.u.) in every simulation block of the calculation biasing only z. It is clearly not converged.

Block	CVs ΔF	z w
1	11.9	640
2	11.1	524
3	7.0	1.4
4	5.6	0.4
5	6.8	5.8
6	-0.3	0.0
7	1.2	0.0
8	3.7	0.2
9	0.8	0.0
all	**11.5** $\pm$ 0.6	

Supplementary Table 3: **Binding free energy breakdown at T=290, 310, 320 and 330 K.** Binding free energy ΔF (kcal mol^{-1}) and its corresponding statistical weight w (a.u.) in every simulation block of calculations biasing z and s_w.

Block	T=290 K		T=310 K		T=320 K		T=330 K	
	ΔF	w	ΔF	w	ΔF	w	ΔF	w
1	5.42	44	6.83	310	5.23	59	6.28	260
2	6.21	110	6.07	200	5.98	120	8.11	270
3	6.70	130	6.01	140	4.25	18	7.14	210
4	5.99	70	5.5	140	5.97	170	5.43	150
5	-	-	6.22	210	6.36	220	5.85	240
6	-	-	6.99	260	7.14	430	5.98	220
all	**6.25** ± 0.26		**6.40** ± 0.24		**6.48** ± 0.40		**6.56** ± 0.42	
N_{eff}	3.5		5.53		3.69		5.82	

Supplementary Table 4: **Binding free energies at different T.**

**T (K)**	290	300	310	320	330
**ΔF (kcal/mol)**	6.25 ± 0.26	6.36 ± 0.07	6.40 ± 0.24	6.48 ± 0.40	6.56 ± 0.42

Supplementary References

[1] Rizzi, V., Bonati, L., Ansari, N. & Parrinello, M. The role of water in host-guest interaction. *Nature Communications* **12**, 93 (2021). URL http://dx.doi.org/10.1038/s41467-020-20310-0http://www.nature.com/articles/s41467-020-20310-0.

[2] Ray, D., Ansari, N., Rizzi, V., Invernizzi, M. & Parrinello, M. Rare Event Kinetics from Adaptive Bias Enhanced Sampling 1–21 (2022). URL http://arxiv.org/abs/2208.04942.

[3] Salvalaglio, M., Tiwary, P. & Parrinello, M. Assessing the Reliability of the Dynamics Reconstructed from Metadynamics. *Journal of Chemical Theory and Computation* **10**, 1420–1425 (2014). URL http://pubs.acs.org/doi/10.1021/ct500040r.

[4] Bonati, L., Rizzi, V. & Parrinello, M. Data-Driven Collective Variables for Enhanced Sampling. *The Journal of Physical Chemistry Letters* **11**, 2998–3004 (2020). URL http://arxiv.org/abs/2002.06562https://pubs.acs.org/doi/10.1021/acs.jpclett.0c00535.

[5] Brotzakis, Z. F. Vac metad applications. https://gitlab.e-cam2020.eu/brotzakis/vac_metad_applications/-/tree/master/TRYPSIN/GROMACS_INPUTe (2019).

[6] Kingma, D. P. & Ba, J. Adam: A Method for Stochastic Optimization. In *ICLR*, 1–15 (2015). URL http://arxiv.org/abs/1412.6980.

[7] Bonati, L., Piccini, G. & Parrinello, M. Deep learning the slow modes for rare events sampling. *Proceedings of the National Academy of Sciences* **118** (2021). URL http://arxiv.org/abs/2107.03943http://www.pnas.org/lookup/doi/10.1073/pnas.2113533118https://pnas.org/doi/full/10.1073/pnas.2113533118.

[8] Roussey, N. M. & Dickson, A. Local Ion Densities can Influence Transition Paths of Molecular Binding. *Frontiers in Molecular Biosciences* **9**, 1–8 (2022). URL https://www.frontiersin.org/articles/10.3389/fmolb.2022.858316/full.

[9] Yin, J., Henriksen, N. M., Slochower, D. R. & Gilson, M. K. The SAMPL5 host–guest challenge: computing binding free energies and enthalpies from explicit solvent simulations by the attach-pull-release (APR) method. *Journal of Computer-Aided Molecular Design* **31**, 133–145 (2017). URL http://link.springer.com/10.1007/s10822-016-9970-8.

[10] Gelenter, M. D. *et al.* Water orientation and dynamics in the closed and open influenza B virus M2 proton channels. *Communications Biology* **4**, 338 (2021). URL http://dx.doi.org/10.1038/s42003-021-01847-2http://www.nature.com/articles/s42003-021-01847-2.
